# Management of Complete Heart Block in a Pregnant Woman with Systemic Lupus Erythematosus-Associated Complications: Treatment Considerations and Pitfalls

**DOI:** 10.3390/medicina59010088

**Published:** 2022-12-31

**Authors:** Eva Rihackova, Petra Vysocanova, Michal Rihacek, Dominika Kucerova, Tereza Blahovcova, Petr Kala

**Affiliations:** 1 Department of Internal Medicine and Cardiology, University Hospital Brno and Faculty of Medicine, Masaryk University, Jihlavska 20, 625 00 Brno, Czech Republic; 2 Department of Laboratory Methods, Faculty of Medicine, Masaryk University, Kamenice 5, 625 00 Brno, Czech Republic; 3 Department of Laboratory Medicine, Masaryk Memorial Cancer Institute, Zluty Kopec 7, 656 53 Brno, Czech Republic; 4 Department of Biochemistry, Faculty of Medicine, Masaryk University, Kamenice 5, 625 00 Brno, Czech Republic; 5 Department of Gynecology and Obstetrics, University Hospital Brno and Faculty of Medicine, Masaryk University, Jihlavska 20, 625 00 Brno, Czech Republic

**Keywords:** complete heart block, lupus erythematosus, pregnancy, methyldopa

## Abstract

We present a case of a pregnant woman with systemic lupus erythematosus (SLE) who was diagnosed with asymptomatic complete heart block (CHB) during pregnancy. To evaluate possible risks and benefits of pacemaker (PM) implantation, a multidisciplinary counselling board was held. Its recommendation was to perform PM implantation to prevent intra-uterine growth restriction from insufficient cardiac output using a fluoroscopic protective shield. The procedure was performed without complications and established permanent pacing on onwards ECG examinations. The patient subsequently gave birth to a healthy newborn. After a retrospective clinical case evaluation and review of relevant literature, a presumptive association between CHB and the primary diagnosis was proposed. Above that, pregnant women with SLE who develop hypertension are commonly treated with methyldopa, which may cause conduction abnormalities. Clinical recommendations for young female patients expecting pregnancy are lacking in this area. Careful diagnostic and treatment approaches should be used in the management of possible SLE-related complications in women of child-bearing age, focusing on preventable events.

## 1. Introduction

Abnormal conduction between the atria and the ventricles is divided into three main degrees (first, second and complete heart block). The first-degree heart block is defined on ECG by a PR interval longer than 0.20 s and is generally asymptomatic [1]. Second-degree heart block is a disease of the cardiac conduction system in which the conduction of atrial impulse through the atrioventricular (AV) node and/or His bundle is delayed or blocked. Patients with second degree heart block may present with wide range of symptoms depending on the severity of conduction defects (e.g., syncope, lightheadedness or no symptoms at all) [2].

Complete heart block (CHB) is characterized by the absence of conduction between the atria and the ventricles. CHB patients most commonly suffer from decreased perfusion, which results in symptoms related to bradycardia. Subsequent low cardiac output can lead to serious arrhythmias such as ventricular tachycardia, syncope or sudden death [3].

CHB can either be congenital or acquired (secondary) [4]. The most common cause of congenital heart block is of autoimmune origin related to the transplacental passage of maternal anti-Ro/SSA or/and anti-La/SSB antibodies with consequent immune-mediated injury to the conduction system [5]. This occurs with 2% of anti-Ro/SSA-exposed pregnancies, and the recurrence rate is nine times higher in subsequent pregnancies [6]. Amongst other causes of congenital heart block, an underlying structural disease is also common [7].

Acquired CHB manifests from coronary artery disease, autoimmune disease, cardiomyopathy, infectious heart disease (myocarditis, Lyme disease, etc.) and as a drug-induced condition, and the latter two causes may possibly be reversible [8,9]. Uncovering the CHB during pregnancy is infrequent. Since CHB is a rare condition in women of child-bearing age, there are only a few case studies published discussing the diagnosis and management of CHB during pregnancy, and thus, no clear guidelines have been established [10,11].

## 2. Case Report and Results

We present a case of 28 year-old woman diagnosed with SLE at the age of 20 years. The diagnosis was made via kidney biopsy due to acute renal failure (creatinine 3.8 mg/dL, blood urea nitrogen 44.88 mg/dL) with severe proteinuria (total protein in urine 7.4 g/day) and edemas preceded by urinary tract infection. Histopathological evaluation showed edematous interstitium without fibrosis, with focal lymphoplasmacellular inflammatory infiltration, enlarged glomeruli with globally enhanced mesangial and endocapillary cellularity and the focal presence of cellular sickles. The presence of huge subendothelial focal immune deposits was noted with Acid Fuchsin Orange G staining. Under electron microscopy, numerous subendothelial immune deposits were confirmed and mesangial changes were present. Initially, her condition was classified as diffuse SLE-related nephritis class IV/A (ISN/RPS,) and her laboratory results met the clinical and laboratory criteria of nephrotic syndrome (serum: albumin 24 g/L, cholesterol 348 mg/dL).

Further clinical signs encompassed SLE-related butterfly rash, pleural and pericardial serous effusions, microcytic normochromic anemia (hemoglobin 111 g/L, MCV 78 fL, MCHC 33.7 g/dL) and positive ANA (titer 1:1600), positive anti-ds DNA and anti-ss DNA antibodies and depleted complement components C3 and C4. Her echocardiography findings, brain MRI and central and peripheral joint examinations were normal. Initial treatment included six series of acute hemodialysis, five series of plasmapheresis and six pulses of cyclophosphamide 750 mg/m^2^ with mesna 400 mg and methylprednisolone 3 g i.v. + oral prednisone in a descending dose. Ovarian suppression was induced with goserelin-acetate 3.6 mg s.c. monthly. No biologics were used during initial treatment. Maintenance therapy included daily azathioprine 2 mg/kg with prednisone 20 mg with which the patient achieved disease remission after 3 years (no clinical signs present, normalized BUN and creatinine values, no proteinuria, negative direct Coombs test). The patient’s ANA titers dropped to 1:100 and anti-ENA decline to negative values. Antibody panel results during treatment are summarized in Table 1. The treatment continued with prednisone 20 mg daily via monotherapy (5 years, slowly reducing the dose to 2.5 mg daily). Prior to the diagnosis of SLE, the patient was otherwise healthy. Primary diagnosis-related comorbidities included secondary anemia, hyperuricemia and frequent UTIs on long-term immunosuppressive treatment, hypertension and hypothyroidism. Prior to pregnancy, the long-term medication consisted of calcitriol 0.5 μg daily, prednisone 2.5 mg once in two days, perindopril 10 mg daily, levothyroxine 25 μg daily, folic acid 10 mg weekly and omeprazole 20 mg once in two days. Her ECG findings were normal, and hypertension was well compensated. However, six months prior to a pregnancy, the patient ceased corticosteroid treatment completely despite the clinical recommendation, which resulted in a subsequent rise in ANA titers.

Early in her 28th year of life, the patient became pregnant and immediately ceased treatment with perindopril, which was swapped with methyldopa 250 mg three times per day. Hydroxychloroquine treatment was recommended to the patient to stabilize primary disease during pregnancy, but that was rejected by the patient. Entry pregnancy maternal anti-Ro/SSA or/and anti-La/SSB antibody screening to evaluate the risk of congenital CHB was negative.

**Figure 1:**
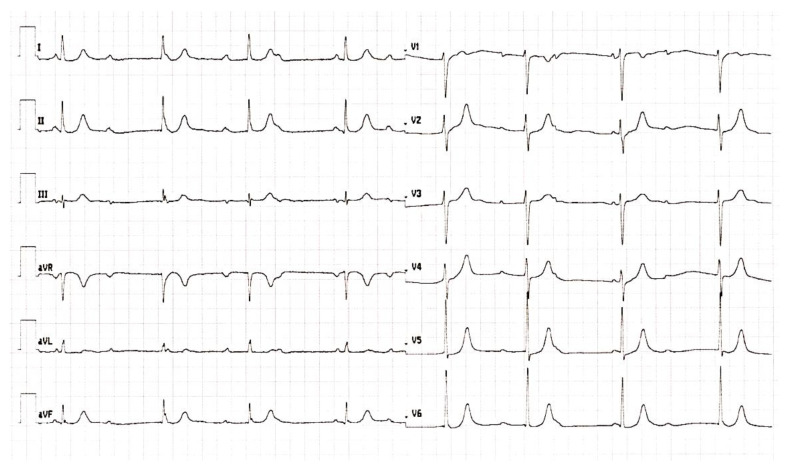
ECG taken at the cardiology department showing the complete heart block.

**Figure 2:**
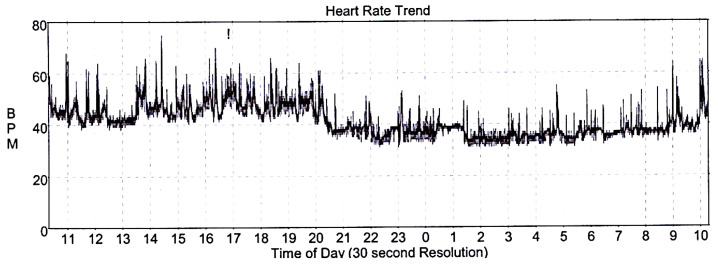
Patient’s ECG 24 h Holter with a mean heart rate of 41 beats per minute and the lowest value of 31 beats per minute.

For insufficient control of blood pressure (BP), the patient had to be referred to a hypertension-treatment specialist for follow-up counselling and treatment re-evaluation during the 8th week of pregnancy. Gradual treatment escalation of methydopa to 500 mg three times per day was introduced with amlodipine 5 mg daily and acetyl-salicylic acid 150 mg daily (for preeclampsia prevention) resulting in adequate BP control. Interestingly, the incidental diagnosis of CHB was incidentally made on the regular ECG check-up (Figure 1), as the patient had not previously experienced any of the common symptoms of low cardiac output, such as fatigue, dizziness, shortness of breath or syncope. The echocardiography showed a normal ejection fraction and no left ventricle dilation and did not discover any valve pathology. Thereafter, the 24 h outpatient ECG Holter exhibited a mean heart rate of 41 beats per minute with the lowest value of 31 beats per minute (Figure 2).

**Figure 3:**
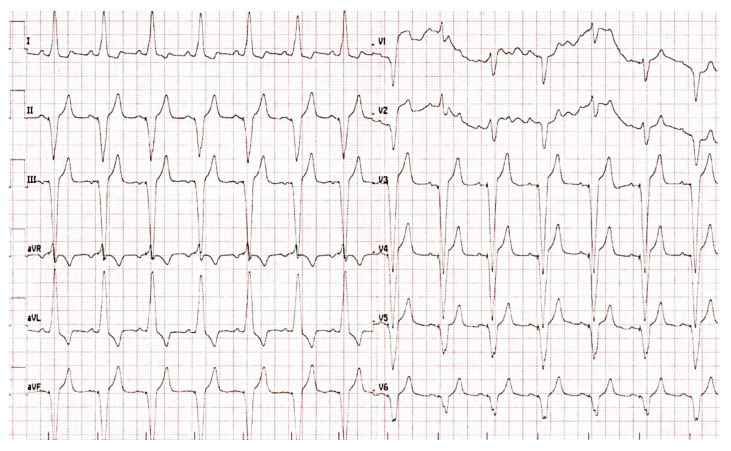
ECG taken based on the routine check-up several months after pacemaker implantation with 100% atrial synchronized ventricular pacing.

The cardiology counselling board with an obstetrician and anesthesiologist evaluated and selected an appropriate treatment approach with regards to available literature. The board agreed to recommend a PM implantation to prevent the impairment of intra-uterine growth restriction from insufficient cardiac output. The Dual-chamber rate-modulated (DDDR) pacemaker (magnetic resonance imaging-compatible) was implanted during the 27th week of pregnancy as demands on the cardiovascular system in this particular pregnancy period are the highest [12]. The procedure was carried out using a protective shield covering the patient’s abdomen without complications. Post-operative ECG showed fully atrial synchronized ventricular pacing (Figure 2). During the 38th week of pregnancy, maternal hepatopathy developed, and therefore, the labor was uncomplicatedly induced. A healthy newborn 48 cm and 2930 g was delivered. On routine cardiology check-up several months afterwards and after methyldopa discontinuation, the pacemaker recording marked 100% atrial synchronized ventricular pacing (Figure 3).

## 3. Discussion

A major association between a congenital CHB in newborns of mothers with SLE-related autoantibodies is well described in current literature [13,14]. In adults with SLE, the manifestation of a complete heart block is a rare cardiac complication, though it is clinically important with possible life-threatening risks, particularly during pregnancy. Current SLE workup protocols include initial echocardiography for screening SLE-related pericarditis or pleural/pericardial effusions; however, no entry-ECG or regular ECG checkups with regular ECG Holter examinations as screening for SLE-related heart conduction abnormalities are neither discussed nor recommended [10,11]. A recent study by Villuendas et al. evaluated a possible link between rheumatologic disease and positive anti-Ro/SSA antibodies, previously discussed as possibly responsible for autoimmune-mediated heart conduction system damage. No association was found, and the results did not support a standard cardiac evaluation in autoimmune disease patients [15]. A study by Natsheh et al. researched case reports of CHB in lupus and found only four anti-Ro/SSA and two anti-La/SSB positive cases out of 32, therefore suggesting other possible pathophysiological mechanisms involved in SLE-induced conduction defects. Additionally, Natsheh et al. discovered that 84% of CHB cases were anti-DNA positive and 100% were ANA positive [16]. Our patient’s anti-Ro/SSA and/or anti-La/SSB antibodies during the pregnancy period were also not elevated but both anti-DNA and ANA were positive. Furthermore, a literature review of CHB cases with lupus erythematosus revealed that CHB almost exclusively involves women (30/32, 94%) and more importantly, the most common CHB manifestation in these women is syncope [16]. Since the probability of preexisting heart disease (such as ischemic heart disease, etc.) was unlikely in our patient, the association between CHB and lupus cannot be neglected. This statement is further supported by the fact that based on follow-up routine pacemaker check-ups, the CHB did not resolve after the discontinuation of methyldopa and the patient continues to have a 100% stimulated PM rhythm.

Recommendations for young women with SLE prior to pregnancy include the multidisciplinary approach of an obstetric and rheumatology team, as disease control (optimally stable disease on no or a minimal dose of oral corticosteroids) is essential for favorable pregnancy outcomes. These recommendations mention possible congenital heart block of the fetus/newborn and its prevention and management; however, no recommendations are aimed towards the possible manifestation of a CHB in the mother [17]. This issue is particularly important as pregnant women with SLE have a higher prevalence of hypertension than those without SLE [18]. Moreover, there have been reports that methyldopa, the drug of choice for pregnant women, may impair atrioventricular conduction and even cause CHB [19,20]. It is assumed that methyldopa, via its central sympatholytic effect, can impair the myocardial conduction system [19]. However, in documented cases, heart conduction defects resolved upon methyldopa discontinuation suggesting reversible impairment of the heart conduction system [19,20].

Although hemodynamic changes during pregnancy are very well known and described (the heart rate increases by 25% and plasma volume increases by more than 1 L) [21] and concomitant CHB with such increased hemodynamic demands may present a significant problem, the relevant clinical guidelines underlining this scenario are still lacking. The inability of the heart to increase the heart rate can affect prenatal development and labor. Cardiac output is based on the stroke volume and heart rate and increases by 20% at the 8th week of gestation to its maximum in the 20–28th week of gestation [12].

Pregnant women with CHB fail to exhibit compensatory heart rate increase, which results in an inadequate hemodynamic status during labor and needs to be corrected by temporary pacing [22]. Hidaka et al. described clinical cases of women with a complete heart block, who received temporary pacing during labor; however, none of them required pacing. According to their experience, it was suggested that women with a complete heart block do not routinely require temporary pacing during labor [23]. This phenomenon could possibly be explained by chronotropic competence in women with CHB during labor, even though this explanation needs to be proven by further studies.

Similarly, Adekanye et al. described a case of a pregnant woman with CHB diagnosed during pregnancy that was left unpaced until labor with temporary pacing equipment on standby. At that time, the pregnant woman was already in her 38th week of pregnancy at which time the growth of the fetus had already finished and the pacemaker was implanted during puerperium [24].

In some cases, decisions about temporary pacemaker insertion during pregnancy have been made based on a clinical history of syncope or presyncope [25]. According to a study from Suri et al., CHB is associated with premature delivery (in three out of four cases) and intrauterine growth restriction (two out of four cases) [26]. With respect to that knowledge, the decision about the implantation of a permanent pacemaker vastly outweighs the risk of intrauterine growth restriction and premature delivery [27]. However, a multidisciplinary approach with a team of cardiologists, obstetricians and anesthetists, as in our case, is essential. Certainly, the decision should consider the gestational week to evaluate the possibility of fetal growth impairment. There are case studies of progressive AV block during pregnancy; therefore, all patients should be carefully monitored [25]. According to a small study by Mandal et al. regarding concomitant CHB and pregnancy outcomes, early-phase pregnancy permanent pacemaker implantation was proposed as a safe and better option to avoid further life-threatening complications [28].

On the contrary, the teratogenic properties of fluoroscopy, routinely used during PM implantation, should not be underestimated. Either a protective shield covering the gravid uterus or a fluoroscopy-free approach using electrophysiologic signals and ECHO guidance for pacemaker insertion should be used [29,30]. Moreover, another modern fluoroscopy-free possibility is to use a cardiac mapping system during the PM implantation procedure [31].

## 4. Conclusions

The management of patients with CHB diagnosed in pregnancy should be performed in tertiary centers. Multidisciplinary evaluation is necessary to select optimal procedures for pregnant women with CHB, as each patient may need an individualized approach.

Due to the risk of intrauterine growth restriction and preterm birth, close fetal and maternal monitoring is essential to provide appropriate care. Women who are diagnosed with SLE are often of childbearing age and may develop CHB both prior to and during pregnancy. According to the study of Tselios et al., the risk of developing a CHB in patients with SLE reaches 1%, while the prevalence of CHB in the general population is 0,04% (including elderly people with pre-existing heart disease) [32,33]. Furthermore, impairments of the heart conduction system may result from concomitant SLE-induced damage, while the use of methyldopa may unmask clinically silent aberrations.

The etiology of lupus-associated complete heart block is unclear, and hence, recommendations are needed. Women with autoimmune disease (especially those with SLE) planning pregnancy are possible candidates for initial 24 h ECG monitoring or for modern wearables for 24 h heart rate monitoring, which may increase the probability of discovering bradycardia, as they have been shown to provide valuable clinical data [34,35]. In patients taking methyldopa or in patients with positive ANA and anti-DNA antibodies, this should be a mandatory clinical measure.

Primary benefits of such diagnostic approaches would be the prevention of severe clinical manifestations, such as syncope, heart failure of the mother and intrauterine growth restriction, fetal impairment and risks associated with PM implantation during pregnancy. Secondary outcomes include the possible elucidation of underlying pathophysiology of CHB in patients with SLE.

## Figures and Tables

**Table 1:** Patient’s antibody panel results and physical parameters during treatment periods.

		Autoantibodies			Physical Parameters	
	ANA	Anti-Ds DNA	Anti-Ss DNA	Anti-ENA	BP (mmHg)	HR (bpm)
At the time of the diagnosis	1:1600	+	+	+/-	180/100	65
Clinically stable disease (3 years on maintenance treatment)	1:100	+	+	-	130/75	70
1 month prior to the PM implantation	1:1600	+	+	-	140/75	40
5 months after the PM implantation	1:3200	+	n/a	-	135/70	85

Aabbreviations: n/a, not available; BP, blood pressure; HR = heart rate; bpm = beats per minute.

## Data Availability

The data presented in this study are available on request from the corresponding author.
